# Role of Mitochondrial Reverse Electron Transport in ROS Signaling: Potential Roles in Health and Disease

**DOI:** 10.3389/fphys.2017.00428

**Published:** 2017-06-27

**Authors:** Filippo Scialò, Daniel J. Fernández-Ayala, Alberto Sanz

**Affiliations:** ^1^Institute for Cell and Molecular Biosciences, Newcastle University Institute for Ageing, Newcastle UniversityNewcastle upon Tyne, United Kingdom; ^2^Centro Andaluz de Biología del Desarrollo, Universidad Pablo de Olavide-CSIC and CIBERER-ISCIIISeville, Spain

**Keywords:** mitochondria, reverse electron transport, ROS, complex I, redox signaling

## Abstract

Reactive Oxygen Species (ROS) can cause oxidative damage and have been proposed to be the main cause of aging and age-related diseases including cancer, diabetes and Parkinson's disease. Accordingly, mitochondria from old individuals have higher levels of ROS. However, ROS also participate in cellular signaling, are instrumental for several physiological processes and boosting ROS levels in model organisms extends lifespan. The current consensus is that low levels of ROS are beneficial, facilitating adaptation to stress via signaling, whereas high levels of ROS are deleterious because they trigger oxidative stress. Based on this model the amount of ROS should determine the physiological effect. However, recent data suggests that the site at which ROS are generated is also instrumental in determining effects on cellular homeostasis. The best example of site-specific ROS signaling is reverse electron transport (RET). RET is produced when electrons from ubiquinol are transferred back to respiratory complex I, reducing NAD+ to NADH. This process generates a significant amount of ROS. RET has been shown to be instrumental for the activation of macrophages in response to bacterial infection, re-organization of the electron transport chain in response to changes in energy supply and adaptation of the carotid body to changes in oxygen levels. In *Drosophila melanogaster*, stimulating RET extends lifespan. Here, we review what is known about RET, as an example of site-specific ROS signaling, and its implications for the field of redox biology.

## Introduction

Mitochondrial reactive oxygen species (mtROS) produced by the respiratory chain (RC) during oxidative phosphorylation are the main source of free radicals in most cell types. The scientific community has changed its opinion of ROS over the last 15 years. Initially ROS were described as simple by-products of metabolism that caused oxidative damage and were responsible for damage upon ischemia-reperfusion or chronic diseases such as Parkinson's or Alzheimer's disease as well as aging (Sanz et al., 2006). However, an increasing amount of evidence has shown that mtROS play a more complex role than previously anticipated, actively participating in cellular signaling. For example hydrogen peroxide (H_2_O_2_), a stable ROS, is able to diffuse far away from the mitochondrion and alter the activity of proteins by regulating the oxidative state of one or more cysteine residues. This type of redox regulation has been shown to be instrumental in controlling many physiological processes (Bak and Weerapana, 2015). Furthermore, several independent reports have shown that boosting mtROS delays aging and the onset of age-related diseases (Hekimi et al., 2011).

The current consensus is that the amount of ROS determines their physiological effects, with low levels of ROS having beneficial effects through stimulation of mitohormesis and high levels causing oxidative damage and contributing to aging. In the last 10 years, the development of new technology and methods has allowed the measurement of mtROS levels in more physiological conditions (Cocheme et al., 2011). Evidence collected using these new techniques indicates that the site at which ROS are generated is as important as the amount of ROS produced.

In mitochondria isolated from rat skeletal muscle 11 different sites at which electrons can leak to produce ROS have been described (Brand, 2016). The amount of ROS produced at each of these sites depends on experimental conditions, including the concentration and combination of substrates used to feed the RC. Experiments performed *in vitro* designed to mimic the cytosolic environment of the muscle (Goncalves et al., 2015) show that the main sites of ROS generation are respiratory complexes I (*aka* NADH:ubiquinone oxidoreductase, CI), II (*aka* succinate-coenzyme Q reductase, CII) and III (*aka* ubiquinol:cytochrome c oxidoreductase, CIII). Unfortunately, there are currently few studies describing how much ROS each respiratory complex produces *in vivo* due to the lack of resolution with these type of measurements (reviewed in Sanz, 2016). Existing data from *in vivo* studies seem to confirm data from *in vitro* work, with a major role for respiratory CI and CIII in ROS production (Chouchani et al., 2014; Scialo et al., 2016). CI generates ROS exclusively into the mitochondrial matrix, while CIII can produce ROS into either the matrix or intermembrane space. The impact of ROS generated at CIII on cellular physiology has been extensively reviewed elsewhere (Sena and Chandel, 2012). For the sake of space, we will focus on ROS produced by CI and particularly on ROS produced via Reverse Electron Transport (RET). Although antioxidant scavenging will modulate the intensity of a ROS signal and the impact on levels of oxidative stress, we will not discuss the role of the several antioxidant systems cells have evolved to prevent oxidative damage and modulate ROS signaling. Instead, we refer readers to some excellent reviews published on this topic (Droge, 2002; Sena and Chandel, 2012).

Until recently, RET was considered as an *in vitro* artifact with no relevance *in vivo*. However, in the last 10 years several groups have independently published evidence that does occur RET *in vivo* and that it has a central role in many physiological processes, including aging. Here, we review what is known about RET *in vivo* including how it is induced and the downstream physiological effects of RET activation.

## How does RET occur *in vivo*?

**Figure 1:**
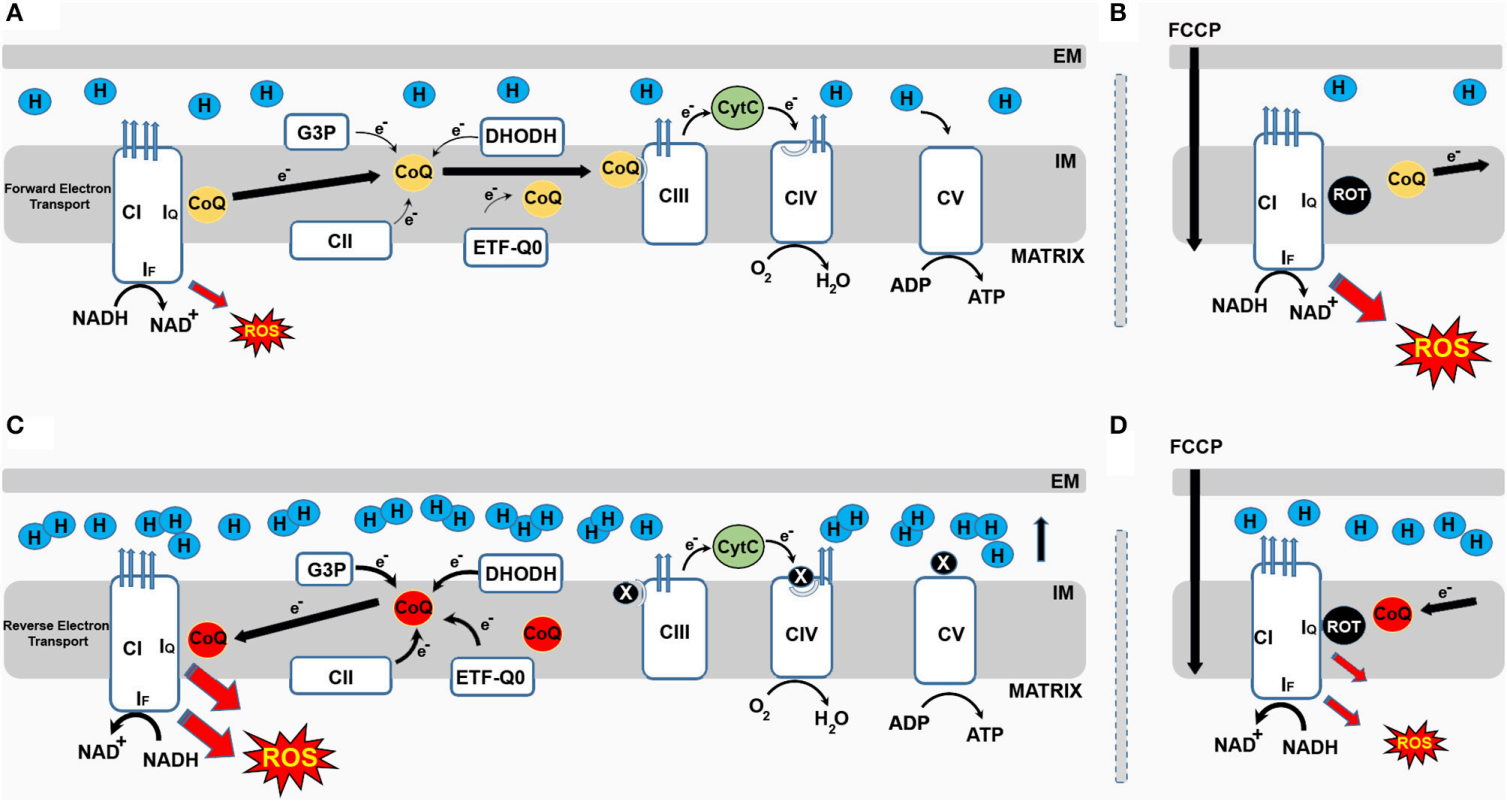
Respiratory CI produces ROS in both the forward and reverse direction. **(A)** During forward electron transfer (FET), CoQ receives electrons from complexes I and II, DHODH, G3P and ETFQO. During this process electrons mainly leak to produce superoxide from the I_F_ site of CI during the oxidation of NADH to NAD+. **(B)** In these conditions, if rotenone blocks the I_Q_ site, electrons cannot be transferred to CoQ, leak and generate ROS. FCCP also increases mtROS generation during FET. **(C)** When the CoQ pool becomes over-reduced, a high membrane potential favors the reverse transfer of electrons from ubiquinol to CI in a process called reverse electron transport (RET). During RET electrons leak at either I_F_ or I_Q_ generating a significant amount of superoxide. **(D)** Blocking the I_Q_ site with rotenone during RET prevents CoQ from transferring electrons back to CI and reduces ROS production. Similarly, FCCP reduces ROS by dissipating membrane potential.

In 1961, Chance and Hollunger (1961) described a previously unknown mechanism by which isolated mitochondria produce NADH from NAD+ after supplying them with succinate (a complex II substrate). Later, it was shown that CI reduces NAD+ to NADH with electrons received from ubiquinol via reverse electron transfer (RET). Interestingly, RET is associated with the generation of high levels of ROS. Now we know that RET occurs when the pool of coenzyme Q (CoQ) becomes over-reduced with electrons from respiratory complex II (*aka* succinate dehydrogenase, CII) (Chouchani et al., 2014). It is possible that other enzymes that introduce electrons downstream of CI such as glycerol-3-phosphate dehydrogenase, electron-transferring flavoprotein (ETF-QO) or dihydroorotate dehydrogenase (DHODH) also contribute to RET generation (Figure 1). Blocking CIII or CIV may also generate conditions for RET. Indirect evidence indicates that nitric oxide inhibits CIV and triggers RET in cell culture (Taylor and Moncada, 2010). However, to the best of our knowledge there is no evidence that this occurs *in vivo*. CoQ plays a major role in mitochondrial respiration since all electrons introduced into the RC will reduce ubiquinone to ubiquinol (Lopez-Lluch et al., 2010). Ubiquinol transfers electrons to CIII becoming re-oxidized to ubiquinone in the process. Since CoQ plays such an essential role, it is not surprising that the most important steps in its synthesis occur within the mitochondrion in a multi-enzyme complex that requires the products of between 10 and 11 genes to be assembled (Bentinger et al., 2010). Mutations in any of these genes can cause severe CoQ deficiency that affects mitochondrial physiology and cellular homeostasis (Fernandez-Ayala et al., 2013). Whether this is caused by alterations in ROS levels remains controversial. In any case, secondary alterations in CoQ synthesis that can alter ROS levels including ROS produced via RET are frequently associated with mitochondrial disease (Fernandez-Ayala et al., 2014).

In addition to a high ratio of ubiquinol to ubiquinone, a high proton motive force (Δp) is required to produce RET. How a sufficiently high Δp is generated or maintained to allow RET *in vivo* remains to be determined. It has been suggested that when RET occurs CV stops producing ATP (Mills et al., 2016). The phosphorylation activity of complex V (CV) could be modulated by ATPIF1. When mitochondrial electron transport is interrupted, CV alters the way it operates, transferring protons from the matrix to the intermembrane space. This process maintains Δp, preventing cell death, but consuming ATP. ATPIF1 inhibits this reverse transfer of protons and thus prevents the depletion of ATP (Esparza-Molto et al., 2017). It has been proposed that ATPIF1 can bind to CV in normal conditions (i.e. when protons flow from the intermembrane space to the matrix) inducing a ROS signal (Esparza-Molto et al., 2017). In these conditions, ROS are probably produced via RET however experimental evidence supporting this is missing. Another possible mechanism for inhibition of CV and induction of RET is via the Krebs' cycle intermediate alpha-ketoglutarate. In a ground-breaking study published in Nature (Chin et al., 2014), Chin and col. demonstrated that alpha-ketoglutarate can inhibit CV and extend lifespan in *Caenorhabidtis elegans*. Although, the mechanism that elicited lifespan extension was not described in detail, we can suggest it may be connected to ROS produced via RET as has been shown in *Drosophila melanogaster* (see below).

## Where are ROS produced within CI?

One important question that needs to be addressed is where electrons leak from to produce ROS within CI (Hirst et al., 2008). Various studies have addressed this question using purified CI, sub-mitochondrial particles or isolated mitochondria. CI, with an approximate size of 1 MDa, is made up of 45 subunits. It has a hydrophobic domain responsible for proton pumping and a hydrophilic domain involved in electron transfer (Zhu et al., 2016). Seven iron-sulfur centers transfer electrons between the flavin mononucleotide (FMN, site I_F_), where NADH is oxidized to NAD+, and the Q-binding site (I_Q_) where electrons are finally transferred to ubiquinone (Figure 1). I_F_, I_Q_ and the iron-sulfur cluster N2 have been proposed to produce superoxide (Scialo et al., 2013).

CI can produce ROS when electrons circulate in the forward or reverse direction. When CI-linked substrates (i.e., glutamate or pyruvate in combination with malate) are used to feed the RC, electrons circulate through CI in the forward direction. In these conditions, blocking the I_Q_ site with rotenone causes accumulation of NADH and over-reduction of the I_F_ site, where electrons leak to reduce oxygen to superoxide (Murphy, 2009). This electron leak and subsequent superoxide formation is independent of both the redox state of CoQ and Δp. When CII-linked substrates (i.e., succinate) are used, a portion of the electrons flow through CI in the reverse direction. ROS produced in these conditions depend on the redox state of CoQ and Δp, with both rotenone and FCCP significantly reducing ROS produced via RET. It is not clear from where electrons leak during RET, although I_Q_ has been proposed as the most probable site of electron leak (Brand et al., 2016).

Understanding where ROS are produced within CI is not only an academic matter. An interesting study from the laboratory of Stefan Drose demonstrated that how ROS are generated (i.e., in forward or reverse direction) at CI affects how several proteins are oxidized (Bleier et al., 2015). This supports the existence of site-specific ROS signaling that activates specific pathways by altering the redox states of specific proteins. This means that stimulating the generation of ROS at specific sites and preventing electron leak from others could have therapeutic effects. Following this logic, Martin Brand's group has started to design antioxidants that target ROS produced exclusively by CI (Brand et al., 2016) or CIII (Orr et al., 2015). In relation to this, we should also discuss rotenone and metformin. Although both rotenone and metformin can inhibit CI, they have very different physiological effects. For example, rotenone exposure causes a Parkinson's like phenotype in animal models, whereas metformin is used to treat diabetes (Celardo et al., 2014). It is not clear where metformin inhibits CI (i.e., I_F_ or I_Q_) or whether its therapeutic properties are related with the inhibition of CI. However, it is possible that the positive effects of metformin are caused by a site-specific inhibition of CI that triggers a specific ROS signal. Given the therapeutic potential of metformin, this possibility should be carefully tested in future. As we will discuss below, ROS produced via RET at CI (RET-ROS) have the potential to be a major signaling pathway between the mitochondrion and the rest of the cell. In normal conditions, this system would contribute to maintaining cellular homeostasis but when dysregulated could result in several pathological effects.

## Reverse electron transport in health

ROS play an essential role in redox signaling (Reczek and Chandel, 2014). As we previously mentioned, the dominant paradigm is that the amount of ROS determines physiological effects. However, in addition to the amount of ROS, the site at which ROS are generated is also important. RET-ROS is particularly well suited to detecting metabolic changes which require metabolic adaptations. RET is sensitive to changes in the redox state of CoQ, which is directly related to electron flow through the RC, and variations in Δp that relate to the capacity to produce ATP. In addition, a considerable amount of ROS is produced from RET. These RET-ROS could act as a signal whose intensity could be modulated.

**Figure 2:**
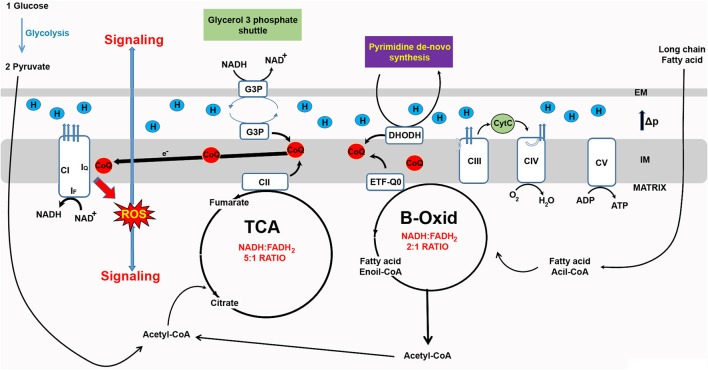
Reverse electron transfer (RET) as a new ROS-based mitochondrial signaling pathway. The redox state of the CoQ pool determines electron leak and superoxide production and can be used by mitochondria as a signaling mechanism. Since RET is sensitive to changes in electron flow and membrane potential, it can be used to detect changes in energy supply and fine-tune cell metabolism. For example, an increase in the availability fatty acid alters the NADH:FADH2 ratio from 5:1 to 2:1 and in response levels of CI are reshuffled by ROS produced via RET (RET-ROS) (Guaras et al., 2016).

In recent years, RET-ROS have been implicated in several physiological processes. Firstly, the differentiation of myoblasts into myotubes requires an increase in mtROS levels which if prevented with antioxidant treatment inhibits differentiation (Lee et al., 2011). Importantly, rotenone, which increases ROS production in the forward direction, but decreases ROS production via RET (Figure 1) also inhibits differentiation (Lee et al., 2011). Similarly, knock down of CI subunits which prevents the assembly of the full complex and decreases RET (Scialo et al., 2016), also reduces myoblast differentiation (Lee et al., 2011). The Enriquez group, at CNIC in Madrid, has recently shown that CI produces ROS in the forward or reverse direction depending on the substrates used to feed the RC. Respiratory complexes are organized in larger superstructures called supercomplexes. Depending on the source of energy available (e.g. sugars or fats), supercomplexes are present in different organizations i.e. CI+CIII+CIV, CI+CIII and CIII+CIV, in addition to CIV alone (Lapuente-Brun et al., 2013). The organization adopted will maximize the use of the available fuel. Oxidation of glucose produces NADH and FADH_2_ with five NADH molecules being oxidized for each molecule of FADH_2_ by the RC. When fatty acids are oxidized the ratio of NADH to FADH_2_ changes to 2:1 which results in an over-reduction of CoQ pool and an increase in RET-ROS (Guaras et al., 2016). In these conditions, RET-ROS promotes the degradation of CI increasing the association between CIII and CIV, which is more efficient for the oxidation of fatty acids. These results resolve many contradictory findings regarding the effect of rotenone on levels of mtROS. As Guaras and col. show (Guaras et al., 2016), rotenone increases ROS when cells are fed with galactose, where ROS are produced in the forward direction by CI, and reduces ROS when cells are fed with fatty acids that stimulate production of RET-ROS (Figure 2).

In response to bacterial infection, macrophages reorganize their RC, decreasing the levels of CI and increasing activity of CII (Garaude et al., 2016). These changes are required to produce an inflammatory response characterized by production of pro-inflammatory cytokines such as interleukin 1β (IL-1β) and IL-10. This suggests that both re-organization of the RC and production of cytokines are elicited via production of RET-ROS. Accordingly, Mills and col. have shown that macrophages alter the way ROS are produced in response to activation by lipopolysaccharide (LPS) (Mills et al., 2016). To achieve this, macrophages interrupt ATP production via oxidative phosphorylation (switching to producing ATP via glycolysis), boost oxidation of succinate by CII and increase mitochondrial membrane potential, subsequently triggering the production of RET-ROS. Suppression of RET-ROS by preventing oxidation of succinate, blocking RET with rotenone or ectopically expressing an alternative oxidase from *Ciona intestinalis* (that prevents the over-reduction of CoQ Scialo et al., 2016) inhibits the generation of pro-inflammatory cytokines required to fight bacterial infection. These results indicate that RET-ROS signaling is instrumental for metabolic and immune reprogramming of macrophages and thus the initiation of an inflammatory response.

Another important physiological phenomenon that seems to be regulated by RET-ROS signaling is the sensing of oxygen levels by the carotid body (CB) (Fernandez-Aguera et al., 2015). The chemoreceptor cells of CB are responsible for inducing a response to hypoxia. Under normoxia, CB cells use NADH and CI produces low levels of ROS in the forward direction. However, under hypoxia, cells undergo a metabolic shift characterized by increased utilization of succinate, which alters ROS production from CI from the forward to the reverse direction (Fernandez-Aguera et al., 2015). In this model, not only ROS but also a boost in NADH levels is required to trigger the hypoxia response, suggesting that RET-ROS signaling may require other signals to elicit a physiological adaptation.

Finally, we have recently showed that induction of RET-ROS in *Drosophila* extends lifespan (Scialo et al., 2016). Accumulation of damage caused by mtROS has been proposed as one of the main drivers of aging (Barja, 2013) although most of the evidence supporting this hypothesis is indirect and based on data from studies using isolated mitochondria (reviewed in Sanz, 2016). Several laboratories have independently shown that increasing ROS in animal models extends lifespan (Sanz, 2016), however the downstream mechanisms that mediate lifespan extension remain to be determined. Ectopic expression of NDI1 from *Saccharomyces cerevisiae* in *D. melanogaster* increases the redox state of CoQ causing a considerable elevation of mtROS levels (Scialo et al., 2016). We observed that ROS were produced via RET at CI, since feeding flies with rotenone or FCCP restored ROS levels to normal. Moreover, co-expression of NDI1 with AOX or knock down of a CI subunit (required for CI assembly) had the same effect. Interestingly, co-expression of NDI1 with a mitochondrially-targeted catalase abolished lifespan extension conferred by NDI1 (Scialo et al., 2016), confirming that lifespan was extended as a result of RET-ROS. Our work is the first to clearly demonstrate that stimulation of RET-ROS *in vivo* can extend lifespan in a complex animal, paving the way for stimulation of RET-ROS production as an anti-aging therapy. In future, we are interested in determining how RET-ROS interact with other signaling pathways that regulate lifespan, such as insulin/insulin-like growth factor/Target of Rapamycin (Scialo et al., 2015), to test whether they extend lifespan via the same or distinct mechanisms.

## Reverse electron transport in disease

As we have discussed, RET-ROS signaling seems to be a newly discovered signaling pathway, instrumental in maintaining cellular and organismal homeostasis. If this is indeed the case, dysregulation of RET-ROS signaling will have pathological consequences. Accordingly, oxidative damage and associated cell death occurring during reperfusion (after an ischemic episode, heart attack or stroke) in heart or brain is caused by an excess of ROS produced via RET (Chouchani et al., 2014). In a seminal study, the group of Mike Murphy, at the MRC Mitochondrial Biology Unit in Cambridge, has shown that succinate accumulates in ischemic tissues (Chouchani et al., 2014). During reperfusion, succinate is rapidly oxidized by CII producing RET-ROS, which cause oxidative damage leading to cell death. Inhibition of CII with dimethyl-malonate or CI with rotenone protects the heart during ischemia-reperfusion. Similarly, a new generation of antioxidants that specifically neutralize ROS produced at the I_Q_ site within CI also alleviate the effects of ischemia-reperfusion in the heart (Brand et al., 2016), suggesting that specific protection against excess RET-ROS will be beneficial in protecting against damage resulting from a heart attack or stroke.

## Conclusion

Mitochondria have evolved several ways to communicate with the other cellular components in order to supply appropriate amounts of energy and essential cellular components (e.g. iron sulfur clusters) produced within the mitochondrion. The entire set of molecules that participate in cellular signaling is not well defined, but a number of transcription factors, RC components, Krebs cycle intermediates and of course, ROS, have been shown to be able to signal from mitochondria. ROS as signaling molecules is particularly complicated since they can cause oxidative damage. It is now widely accepted that ROS participate in signaling at low levels, but cause damage at higher concentrations. However, new evidence indicates that the site at which ROS are generated is also important in determining the effects of ROS. In fact, it follows logic that ROS involved in signaling are produced in specific locations at specific times. This pathway would rely on redox sensitive proteins, located in proximity to the site of ROS production to relay complex information. From this point of view, RET-ROS signaling is an ideal system, since it detects changes in membrane potential and electron flow, producing significant amount of ROS exclusively at CI. A number of independent reports support a role for RET-ROS signaling in physiological and pathological situations and even in the regulation of lifespan. Further investigation using genetic tools and *in vivo* imaging will help to determine the importance of RET-ROS signaling in health and disease.

## Author contributions

All authors listed, have made substantial, direct and intellectual contribution to the work, and approved it for publication.

### Conflict of interest statement

The authors declare that the research was conducted in the absence of any commercial or financial relationships that could be construed as a potential conflict of interest.
